# Routes to cancer diagnosis for patients with pre-existing psychiatric disorders: a nationwide register-based cohort study

**DOI:** 10.1186/s12885-022-09598-x

**Published:** 2022-04-29

**Authors:** Line Flytkjær Virgilsen, Peter Vedsted, Alina Zalounina Falborg, Anette Fischer Pedersen, Anders Prior, Henry Jensen

**Affiliations:** 1 Research Unit for General Practice, Bartholins Alle 2, 8000 Aarhus, Denmark; 2 Department of Clinical Medicine, Aarhus University, Palle Juul-Jensens Boulevard 82, 8200 Aarhus C, Denmark

**Keywords:** (MeSH): Denmark, Neoplasms, Early detection of Cancer, Mental disorders

## Abstract

**Background:**

Poor cancer prognosis has been observed in patients with pre-existing psychiatric disorders. Therefore, we need better knowledge about the diagnosis of cancer in this patient group. The aim of the study was to describe the routes to cancer diagnosis in patients with pre-existing psychiatric disorders and to analyse how cancer type modified the routes.

**Methods:**

A register-based cohort study was conducted by including patients diagnosed with incident cancer in 2014–2018 (*n* = 155,851). Information on pre-existing psychiatric disorders was obtained from register data on hospital contacts and prescription medication. Multinomial regression models with marginal means expressed as probabilities were used to assess the association between pre-existing psychiatric disorders and routes to diagnosis.

**Results:**

Compared to patients with no psychiatric disorders, the population with a psychiatric disorder had an 8.0% lower probability of being diagnosed through cancer patient pathways initiated in primary care and a 7.6% higher probability of being diagnosed through unplanned admissions. Patients with pre-existing psychiatric disorders diagnosed with rectal, colon, pancreatic, liver or lung cancer and patients with schizophrenia and organic disorders were less often diagnosed through cancer patient pathways initiated in primary care.

**Conclusion:**

Patients with pre-existing psychiatric disorders were less likely to be diagnosed through Cancer Patient Pathways from primary care. To some extent, this was more pronounced among patients with cancer types that often present with vague or unspecific symptoms and among patients with severe psychiatric disorders. Targeting the routes by which patients with psychiatric disorders are diagnosed, may be one way to improve the prognosis among this group of patients.

**Supplementary Information:**

The online version contains supplementary material available at 10.1186/s12885-022-09598-x.

## Background

Each year, more than 40,000 Danes are diagnosed with cancer which also is the leading cause of death in Denmark [1, 2]. The prognosis after cancer has previously been reported to be strongly associated with the patient’s Route to Diagnosis (RtD). Across cancer types, patients presenting through an emergency route have displayed inferior prognosis compared to patients beginning their diagnostic trajectory in primary care, e.g. through urgent referrals to secondary care like the two-week wait (TWW) in the United Kingdom or a Cancer Patient Pathway (CPP) in Scandinavia [3, 4]. In Denmark, more than 75% of all cancer patients begin the diagnostic process by presenting symptoms in primary care [5], and 45% are diagnosed after referral to a CPP [6].

The prevalence of psychiatric disorders is around 8–30% in the general population, depending on the included disorders and timeframe [7–9]. Patients with psychiatric disorders have a reduced life expectancy of up to 20 years compared to the general population [10, 11]. This excess mortality has been linked with several factors, e.g. lifestyle, social issues, adverse medication effects, healthcare provision, suicide risk and comorbidity [10, 12, 13] and challenges have also been reported across the entire spectrum of cancer care, from symptom presentation to end-of-life care [13–16]. Inferior cancer prognosis has been reported in patients with psychiatric disorders [14, 17–19] and suboptimal diagnostic process in this patient group has been highlighted as a possible explanation [10, 20] including underdetection, delayed diagnosis and lower screening uptake [14, 21–24]. To our knowledge, only limited research has explored the RtD among cancer patients with pre-existing psychiatric disorders [25].

Further attention is warranted on the diagnostic process in cancer patients with psychiatric disorders [10, 14]. A starting point could be to study how this patient group enters the diagnostic pathway, and whether specific subgroups are particularly challenged by less favourable diagnostic trajectories (defined here as lower referral to primary care initiated CPP and higher rates of unplanned admissions as this have been linked with worse prognosis after cancer [3, 6]). We hypothesised that patients with pre-existing psychiatric disorders were more often diagnosed through less favourable routes than patients without psychiatric disorders. We also hypothesised that some subgroups of patients with psychiatric disorders, e.g. schizophrenia, would more often present through unplanned admission compared to patients with other subtypes of psychiatric disorders. Finally, we hypothesised that the association between pre-existing psychiatric disorders and RtD would vary between cancer types.

## Methods

### Aim and design

The aim of the study was to describe the Routes to Diagnosis of cancer among patients with pre-existing psychiatric disorders and to analyse how cancer type modified the routes. This was studied by conducting a national cohort study using national register-based data in Denmark.

### Setting

Denmark has a population of 5.8 million inhabitants, and 98% of the population is listed with a general practice clinic. The healthcare system is based on free and equal access to most medical services, which are funded by tax revenues. The general practitioner (GP) serves as gatekeeper to secondary care [26] and can refer patients to one of the 30 existing CPPs covering approximately 40 different cancer diseases implemented between 2008 and 2010. If relevant, the GP can also refer to other diagnostic services at the hospital or at private specialist clinics. National screening programmes exist for cervical cancer, colorectal cancer and breast cancer [27, 28].

### Study population

The study population was defined as patients aged 18 years or older with a first-time cancer, excluding non-melanoma skin cancer (International Classification of Diseases, 10th version (ICD-10): C44), recorded in the Danish Cancer Registry [29] between 1 January 2014 and 31 December 2018. In total, 159,189 patients were identified. The final study population comprised 155,851 patients (97.9%) after exclusion of patients with no registration on sex (*n* = 1391) or age (*n* = 798), patients who had migrated within 10 years prior to the cancer diagnosis (*n* = 534), patients registered with multiple cancer types on the same date of diagnosis (*n* = 446) and male patients with breast cancer (as no specific route could be assigned to this group) (*n* = 169).

### Data collection

The data was collected from the national registers and clinical databases described below. We used the unique civil registration number assigned to all Danish citizens to link data between registers at the individual level [30].

### Definition of routes to diagnosis (RtD)

Danish registers have no systematically collected data on RtD for cancer. Thus, an algorithm developed by Danckert B et al. [6] was used to assign each included patient with a RtD. The algorithm used national register data to identify the most likely RtD based on the patient’s contacts in the healthcare system in the time leading up to the cancer diagnosis. The following registers provided the basis for the defined RtD. *The Danish Cancer Registry* provided information on cancer type and whether the patient was diagnosed by death certificate only (DCO). *The Danish National Patient Registry (DNPR)* [31] provided data on all contacts with somatic hospitals in Denmark, including information on inpatient and outpatient visits, dates, department codes and CPP registrations from both primary and secondary care (both from the private and public sector). Three clinical databases *the Danish Breast Cancer Group* [32], *the Danish Colorectal Cancer Database* [33] and *the Danish Quality Database for Cervical Cancer Screening* [34], provided information on screening for breast, colorectal and cervical cancer. Finally, the Danish Register of Causes of Death [35] provided information of vital status and date of death.

Eight mutually exclusive routes were identified: 1) *death certificate only* (DCO) in which only the death certificate provided information on the diagnosis, 2) *screening* in which the patient was diagnosed with breast cancer, colorectal cancer or cervical cancer detected in a national screening programme, 3) *CPP – primary care* in which the patient was diagnosed after CPP referral by a healthcare professional in primary care within 90 days of the diagnosis and no registration of screening detection, 4) *CPP – secondary care* in which the patient was diagnosed after CPP referral by a healthcare professional in secondary care, such as a medical specialist in a hospital within 90 days of the diagnosis and no previous registration of CPP from primary care or screening detection, 5) *unplanned admission* in which the patient had an inpatient hospital admission coded as acute within 30 days before the cancer diagnosis and no prior CPP initiation or screening detection, i.e. comprising both patients admitted through emergency and patients admissions which was not scheduled or planned, 6) *planned admission (elective)* for other reasons than cancer within 30 days before the cancer diagnosis and no prior CPP initiation or screening detection, 7) *outpatient visit* (outpatient hospital specialist clinic) within 30 days before the cancer diagnosis and no prior CPP initiation, planned admission or screening detection and 8) *unknown* when no specific route could be assigned to the patient based on the existing data.

### Definition of pre-existing psychiatric disorders

Data on pre-existing psychiatric disorders was obtained from *the Danish Psychiatric Central Research Register* (PCRR), which holds information on inpatient, outpatient and emergency contacts to all psychiatric hospitals in Denmark since 1970 [36]. To ensure inclusion of all registered psychiatric disorders, diagnosis codes were also searched in the DNPR. The following groups of pre-existing psychiatric disorders were included: organic disorders (ICD-10: F00-F09), substance use disorders (ICD-10: F10-F19), schizophrenia and psychotic disorders (ICD-10: F20-F29), mood disorders (ICD-10: F30-F39), anxiety disorders (ICD-10: F40-F41) and stress disorders (ICD-10: F43). Further, data on prescription medication was obtained from *the Danish National Prescription Registry* [37]. We included the variable “prescription-based mental disorders”, which was defined as individuals who had been prescribed antidepressants (Anatomical Therapeutic Chemical (ATC): N06A), anxiolytics (ATC: N05B) or antipsychotics (ATC: N05A) at least two times within 1 year of the cancer diagnosis date and had *no* registrations in any of the other included subgroups. This subgroup was included to describe patients with pre-existing psychiatric disorders treated mainly in primary care.

### Covariates

The following variables were assessed as potential confounders. Sex and age were obtained from *the Danish Civil Registration System*. Age was categorised in five groups: 18–49, 50–59, 60–69, 70–79 and 80+ years. The patient’s highest attained education in the year of diagnosis was defined according to the International Standard Classification of Education (ISCED) [38] and divided into short (≤10 years), medium (11–15 years) and long education (> 15 years). Ethnicity was divided into Danish and immigrant. Marital status was divided into married/cohabiting and living alone. Year of diagnosis and cancer type were defined based on registrations in *the Danish Cancer Registry*, and 23 types of cancer were defined (see Additional file 1 for overview). The patient’s burden of comorbidity was assessed by means of the Charlson’s Comorbidity Index (CCI) [39] (excluding cancer) based on diagnosis registrations in the DNPR for up to 10 years prior to the diagnosis. Comorbidity burden was categorised into none (CCI score of 0), low (CCI score of 1–2) and high (CCI score of ≥3). Region of residence was based on the five geographically defined areas in Denmark.

### Statistical analyses

A descriptive overview of the RtD was provided by assessing the proportion of RtD, including 95% confidence intervals (CI), according to pre-existing psychiatric disorders and subgroups of psychiatric disorders.

Multinomial logistic regressions (MLRs) were performed to assess the RtD for patients with psychiatric disorders compared to those without. An unadjusted model is presented and followed by a model adjusted for sex, age, CCI, ethnicity, marital status, education, year of diagnosis, ethnicity and region of residence. Cluster robust standard errors were used to account for the effect of clustering of observations around cancer diagnoses. The results are presented as relative risk ratios (RRRs) with “CPP – primary care “as the reference (Table 3).

**Fig. 1:**
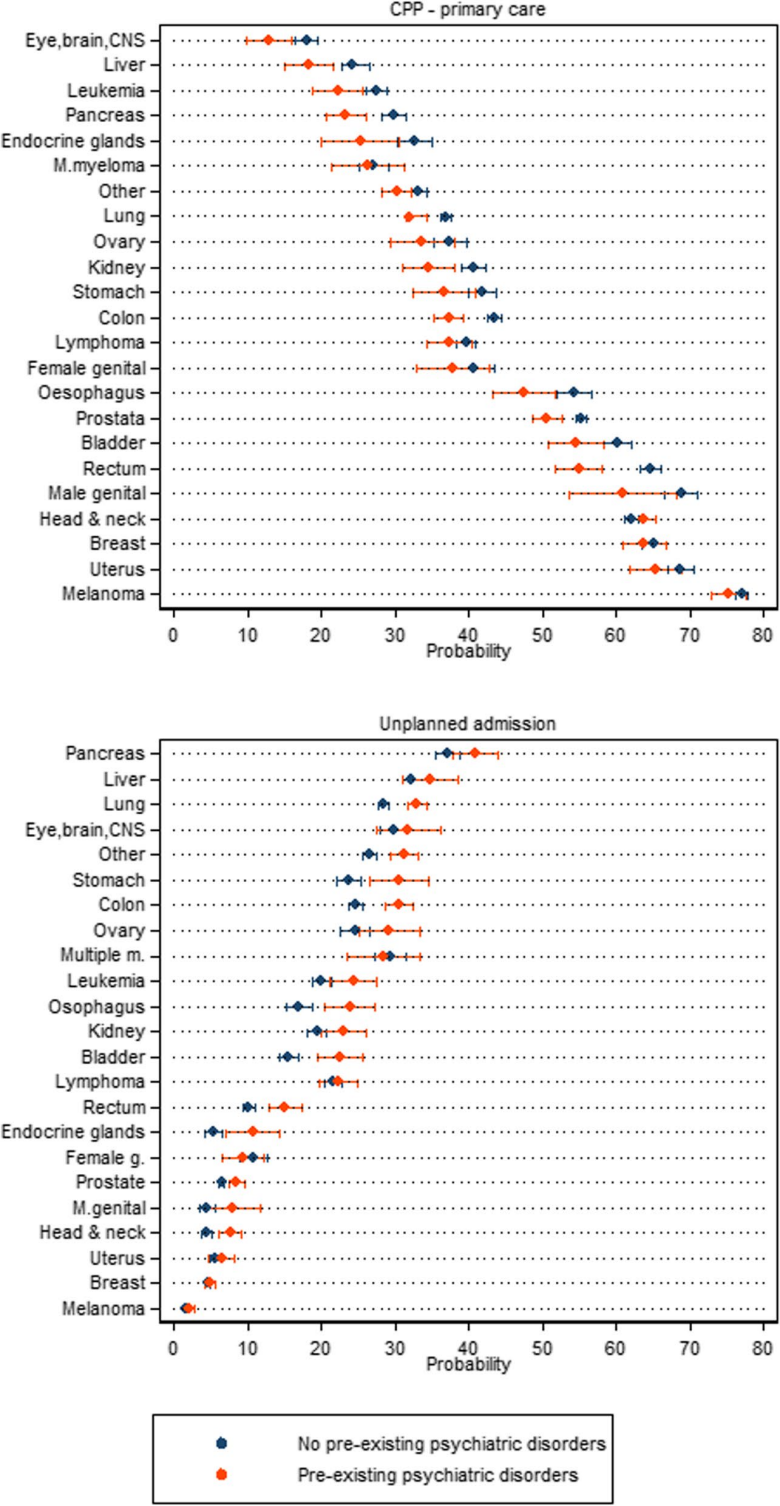
Estimated* probabilities of patients presenting through a CPP in primary care and unplanned admission for each cancer type according to any pre-existing psychiatric disorders. *Adjusted for sex, age, year of diagnosis, comorbidity, education, ethnicity, cohabitation and region of residence

**Fig. 2:**
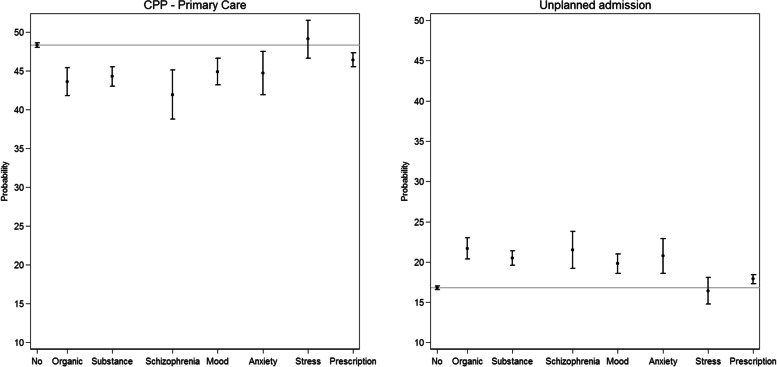
Estimated* probabilities of patients presenting through CPP - primary care and unplanned admission according to subgroups of pre-existing psychiatric disorders. Abbreviation: no = no pre-existing psychiatric disorder, schizophrenia = schizophrenia and psychotic disorders, Prescription: prescription-based mental disorders. *Adjusted for sex, age, year of diagnosis, comorbidity, education, ethnicity, cohabitation, region of residence, cancer diagnosis and for each of the other subgroups of pre-existing psychiatric disorders. The grey line represents the probability of each route for patients with no pre-existing psychiatric disorders

Stratified MLRs were conducted after an interaction test between cancer type, pre-existing psychiatric disorders and RtD. The cancer-specific analyses (Fig. 1) and the analyses based on subtypes of psychiatric disorders (Fig. 2) focused on two outcomes only: “CPP – primary care” and “unplanned admission”. They were chosen as the largest absolute difference in having a pre-existing psychiatric disorder were observed for these routes and because they also represented the poorest and the best prognosis among symptomatic cancer types [6]. The results are expressed as probabilities and are based on marginal means, which were computed with covariates at their observed value. The models were fully adjusted as described above, where each analysis for a specific subgroup of psychiatric disorders was additionally adjusted for the other subgroups of psychiatric disorders due to the risk of psychiatric comorbidity [40] . Robust variance estimates were included in all stratified analyses to allow for clustering of patients by general practice.

Sensitivity analyses were conducted when 1) the pre-existing psychiatric disorders included patients registered in the PCRR and the DNPR within 2 years of the diagnosis (instead of 5 years) and expanding to 10 year prior to the diagnosis, when 2) the assessment of psychiatric disorders were based on only registrations in the PCRR and the DNPR at 5 years prior the diagnosis, thus excluding the category “prescription based mental disorders”, and when 3) the analyses stratified on cancer type were also conducted without prescriptions as an indication for pre-existing psychiatric disorder.

## Results

### Descriptive data

The study population had a mean age of 66 years, and 48.5% were female. In total, 18.1% were registered with a pre-existing psychiatric disorder. The most common category was prescription-based mental disorders, which was followed by substance abuse disorders. The most common RtD was CPP through primary care and the rarest group was DCO. Unplanned admission comprised 15.5% of the routes (Table 1). In total, 82.7% of the unplanned admissions were due to unscheduled admission and 17.2% was due to emergency which was similar for patients with and without psychiatric disorders (18.0% vs 17.0%) (data not shown). The most common cancer types were breast, prostate and lung cancer (Table 1) (Additional file 1, part 1).

**Table 1:** Characteristics of included cancer patients (*n* = 155,851)

	Patients without pre-existing psychiatric disorders		Patients with pre-existing psychiatric disorders^a^		Total	
**Total, n (%)**	127,702	(100.0)	28,149	(100.0)	155,851	(100.0)
**Age at diagnosis, mean (SD)**	66.6	(13.2)	66.2	(14.1)	66.53	(13.30)
**Sex, n (%)**						
Female	59,807	(46.8)	15,838	(56.3)	75,645	(48.5)
Male	67,895	(53.2)	12,311	(43.7)	80,206	(51.5)
**Subgroups of pre-existing psychiatric disorders**^a^**, n (%)**						
Mood disorders	–	–	4078	(14.5)	4078	(14.5)
Anxiety disorders	–	–	1258	(4.5)	1258	(4.5)
Stress disorders	–	–	1815	(6.4)	1815	(6.4)
Substance abuse disorders	–	–	6558	(23.3)	6558	(423.3)
Schizophrenia and psychotic disorders	–	–	985	(3.5)	985	(3.5)
Organic disorders	–	–	3102	(11.0)	3102	(11.0)
Prescription-based mental disorders^b^	–	–	14,459	(51.4)	14,459	(51.4)
**Marital status, n (%)**						
Married or cohabiting	78,087	(61.3)	12,882	(45.8)	90,969	(58.5)
Living alone	49,397	(38.7)	15,254	(54.2)	64,651	(41.5)
**Ethnicity, n (%)**						
Danish	121,052	(94.8)	26,701	(94.9)	147,753	(94.8)
Immigrants	6650	(5.2)	1448	(5.1)	8098	(5.2)
**Education, n (%)**						
Short	42,238	(33.2)	12,424	(44.2)	54,662	(35.2)
Medium	59,388	(46.7)	11,613	(41.3)	71,001	(45.7)
Long	25,569	(20.1)	4062	(14.5)	29,631	(19.1)
**Cancer type (details in Additional file****1****, part 1), n (%)**						
Breast	19,137	(14.2)	4044	(14.4)	22,181	(14.2)
Colon	12,487	(9.8)	2615	(9.3)	15,102	(9.7)
Rectum	6145	(4.8)	1086	(3.9)	7231	(4.6)
Lung	14,679	(11.5)	4803	(17.1)	19,482	(12.5)
Prostate	18,137	(14.5)	2353	(8.4)	20,846	(13.4)
Malignant melanoma	9724	(7.6)	1368	(4.9)	11,092	(7.1)
**Year of diagnosis, n (%)**						
2014	25,609	(20.1)	5790	(20.6)	31,399	(20.1)
2015	25,160	(19.7)	5785	(20.6	30,945	(19.9)
2016	25,584	(20.0	5674	(20.2)	31,258	(20.1)
2017	25,632	(20.1)	5587	(19.8)	31,219	(20.0)
2018	25,717	(20.1)	5313	(18.9)	31,030	(19.9)
**Charlson Comorbidity Index score, n (%)**						
None	82,202	(64.4.9)	12,375	(44.0)	94,577	(60.7)
Low (CCI: 1–2)	33,829	(26.5)	10,205	(36.3)	44,034	(28.3)
High (CCI: ≥3)	11,671	(9.1)	5569	(19.8)	17,240	(11.1)
**Route to diagnosis**						
DCO	373	(0.3)	420	(1.5)	793	(0.5)
Screening	10,181	(8.0)	1724	(6.1)	11,905	(7.6)
CPP primary care	58,310	(45.7)	10,619	(37.7)	68,929	(44.2)
CPP secondary care	23,941	(18.7)	6128	(21.8)	29,727	(15.5)
Unplanned admissions	18,095	(14.2)	6128	(21.8)	24,223	(15.5)
Elective - others	1415	(1.1)	392	(1.4)	1843	(1.2)
Outpatient – others	8945	(7.0)	1831	(6.5)	10,776	(6.9)
Unknown	6406	(5.0)	1249	(4.4)	7655	(4.9)

*Abbreviations*: *n* number, *SD* standard deviation

^a^comprises persons with at least one of the included diagnoses for up to 5 years prior to the cancer diagnosis or at least two prescriptions of medication to treat psychiatric disorders during 1 year prior to the cancer diagnosis. As some persons were registered with several diagnosis, the cumulative addition of the subgroups exceed the total number of persons with pre-existing psychiatric disorders

^b^defined based on prescription of antidepressant, anxiolytic and antipsychotic medicine, but no registered diagnosis in the hospital sector

### RtD among patients with pre-existing psychiatric disorders

Table 2 presents the proportions of RtD according to pre-existing psychiatric disorders. Diagnosis through a CPP initiated in primary care was seen for 45.7% for patients without and for 37.7% for patients with psychiatric disorders; the lowest percentage was observed for patients with organic disorders (31.9%). The proportion of diagnoses following unplanned admission was highest for patients with organic disorders (31.5%) and lowest for patients without psychiatric disorders (14.2%).

**Table 2:** Proportions of patients presenting in each RtD according to pre-existing psychiatric disorders and subgroups with 95% CI (*n* = 155,851)

	Routes to Diagnosis															
	DCO		Screening		CPP – primary care		CPP – secondary care		Unplanned admission		Elective – other		Outpatient – other		Unknown	
	%	95% CI	%	95% CI	%	95% CI	%	95% CI	%	95% CI	%	95% CI	%	95% CI	%	95% CI
**Psychiatric disorders (any)**																
No (*n* = 127,702)	0.3	(0.3–0.3)	7.9	(7.8–8.1)	45.7	(45.3–45.9)	18.7	(18.5–19.0)	14.2	(14.0–14.4)	1.1	(1.0–1.2)	7.0	(6.9–7.1)	5.0	(4.9–5.1)
Yes (*n* = 28,149^a^)	1.5	(1.4–1.6)	6.1	(5.9–6.4)	37.7	(37.1–38.3)	20.6	(20.1–21.0)	21.8	(21.3–22.2)	1.4	(1.3–1.5)	6.5	(6.2–6.8)	4.4	(4.2–4.7)
Mood disorders																
Yes (*n* = 4078)	1.4	(1.1–1.8)	6.6	(5.9–7.4)	36.2	(34.7–37.7)	19.7	(18.5–20.9)	23.2	(21.9–24.5)	1.6	(1.3–2.0)	7.0	(6.3–7.8)	4.4	(3.8–5.1)
Anxiety disorders																
Yes(*n* = 1258)	4.4	(3.4–5.6)	7.5	(6.1–9.1)	33.7	(31.1–36.3)	18.5	(16.5–20.8)	23.1	(20.8–25.5)	1.7	(1.1–2.5)	6.3	(5.1–7.8)	4.9	(3.9–6.3)
Stress disorders																
Yes (*n* = 1815)	0.8	(0.5–1.4)	7.9	(6.8–9.3)	39.3	(37.1–41.6)	21.7	(20.0–23.7)	18.3	(16.6–20.1)	2.1	(1.6–2.9)	6.1	(5.1–7.3)	3.6	(2.9–4.6)
Substance use disorders																
Yes (*n* = 6558)	0.9	(0.7–1.2)	5.4	(5.0–6.0)	35.0	(33.8–36.2)	21.5	(20.5–22.5)	25.8	(24.7–26.8)	1.5	(1.2–1.8)	5.7	(5.2–6.3)	4.2	(3.7–4.7)
Schizophrenia and psychotic disorders																
Yes (*n* = 985)	1.4	(0.8–2.4)	6.1	(4.8–7.8)	32.8	(30.0–35.8)	20.5	(18.1–23.1)	26.0	(23.3–28.8)	1.2	(0.7–2.1)	6.5	(5.1–8.2)	5.5	(4.2–8.2)
Organic disorders																
Yes (*n* = 3102)	3.4	(2.8–4.0)	1.7	(1.3–2.2)	31.9	(30.2–33.5)	17.7	(16.4–19.1)	31.5	(29.1–33.2)	1.5	(1.0–1.9)	7.2	(6.3–8.2)	5.2	(4.5–6.1)
Prescription-based mental disorders																
Yes (*n* = 14,459)	1.2	(1.1–1.5)	6.7	(6.3–7.1)	40.2	(39.4–41.0)	20.8	(20.2–21.5)	18.8	(18.2–19.4)	1.2	(1.1–1.4)	6.6	(6.2–7.0)	4.4	(4.0–4.7)

*Abbreviations*: *CI* confidence interval, *CPP* cancer patient pathway, *DCO* death certificate only

^a^comprises persons with at least one of the included diagnoses for up to 5 years prior to the cancer diagnosis or at least two prescriptions of medication to treat psychiatric disorders during 1 year prior to the cancer diagnosis. As some persons were registered with several diagnosis, the cumulative addition of the subgroups exceed the total number of persons with pre-existing psychiatric disorders

Patients with psychiatric disorders had higher risk of being diagnosed with cancer through DCO, CPP initiated in secondary care, unplanned admission, elective and outpatient care (relative to a CPP initiated in primary care) compared to the corresponding risk in patients without psychiatric disorders (Table 3). For example, patients with psychiatric disorders had a higher risk of presenting through an unplanned admission (relative to a CPP initiated in primary care) compared to patients without psychiatric disorders (RRR: 1.41, 95% CI 1.28–1.55). These estimates were largely comparable across different definitions of pre-existing psychiatric disorders (Additional file 1, part 3).

**Table 3:** Relative risk ratio for presenting in each route compared to presenting through a CPP in primary care according to any pre-existing psychiatric disorders

			Unadjusted		Adjusted ^a^	
	N	(%)	RRR	95% CI	RRR	95% CI
**DCO**						
Any psychiatric disorder						
No	373	(47.0)	1		1	
Yes	420	(53.0)	**6.18**	**(5.00–7.65)**	**3.54**	**(2.96–4.24)**
**Screening**						
Any psychiatric disorder						
No	10,181	(85.5)	1		1	
Yes	1724	(14.5)	0.93	(0.73–1.18)	0.90	(0.79–1.02)
**CPP** – **primary care**						
Any psychiatric disorder						
No	58,310	(84.6)	**–**	**–**	**–**	**–**
Yes	10,619	(15.4)	**–**	**–**	**–**	**–**
**CPP** – **secondary care**						
Any psychiatric disorder						
No	23,941	(80.5)	**1**		**1**	
Yes	5786	(19.5)	**1.33**	**(1.11–1.59)**	**1.14**	**(1.03–1.27)**
**Unplanned admission**						
Any psychiatric disorder						
No	18,095	(74.7)	**1**		**1**	
Yes	6128	(25.3)	**1.86**	**(1.58–2.19)**	**1.41**	**(1.28–1.55)**
**Elective – other**						
Any psychiatric disorder						
No	1451	(78.8)	**1**		1	
Yes	392	(21.2)	**1.48**	**(1.18–1.86)**	**1.24**	**(1.03–1.49)**
**Outpatient – other**						
Any psychiatric disorder						
No	8945	(83.0)	1		**1**	
Yes	1831	(17.0)	1.12	(0.99–1.27)	**1.09**	**(1.00–1.17)**
**Unknown – other**						
Any psychiatric disorder						
No	6406	(83.7)	1		1	
Yes	1249	(16.3)	1.07	(0.83–1.39)	1.16	(0.97–1.36)

*Abbreviations*: *CI* confidence interval, *CPP* Cancer Patient Pathway, *DCO* Death Certificate Only, *RRR* relative risk ratio

^a^Adjusted for sex, age, year of diagnosis, comorbidity, education, ethnicity, cohabitation, region of residence and cancer diagnosis

### Cancer type and RtD for patients with pre-existing psychiatric disorders

A test for interaction showed that the association between psychiatric disorders and RtD was statistically different across cancer types (*p* < 0.001). No major difference could be observed in the probability to be diagnosed through a CPP initiated in primary care between patients with and without psychiatric disorders for breast, melanoma, uterus, lymphoma, multiple myeloma, and head and neck cancer. In the sensitivity analysis, excluding prescription medication, the results were comparable, although with accentuated associations (Additional file 1, part 4).

### Subtypes of pre-existing psychiatric disorders and routes to diagnosis

The probability of being diagnosed through a CPP initiated in primary care or an unplanned admission varied for subtypes of psychiatric disorders (Fig. 2). The lowest probability of being diagnosed through a CPP initiated in primary care was observed among patients with schizophrenia (41.9, 95% CI: 38.8–45.1) and patients with organic disorders (43.6, 95% CI: 41.8–45.4). These two groups also had the highest probability of being diagnosed after an unplanned admission. Patients with stress disorders had a higher probability (although not statistically significant) of being diagnosed after a CPP referral from primary care compared to patients with no psychiatric disorders also after adjustments of other psychiatric disorders.

## Discussion

### Main findings

Patients with pre-existing psychiatric disorders had a statistically significantly lower probability of being diagnosed through a CPP initiated in primary care and a higher probability of being diagnosed after an unplanned admission compared to patients without psychiatric disorders. This was most pronounced for patients with rectal, colon, pancreatic, liver or lung cancer and for patients with schizophrenia or organic disorders. The RtD did not differ for patients with stress disorders compared to those with no psychiatric disorders, and no differences were seen for breast and melanoma cancer.

### Strengths and limitations

The main strengths of this study was the population-based design and the use of national registers from both the public and private sector with high data validity and almost complete coverage of the Danish population [29, 31, 41]. This minimised the risk of selection bias and information bias. The algorithm for RtD was developed in an international collaboration and modified to fit the Danish healthcare system. Comparing the Danish algorithm to data from England, the Danish algorithm performed similar with expected variations across cancer types (see also [6]). Embedded timeframes constitute an assumption of the algorithm, e.g. information on CPP referral was assessed for up to 90 days before a diagnosis. Patients with psychiatric disorders may be more likely to experience long diagnostic intervals [23] and thus more susceptible to misclassification by the algorithm, e.g. by initiation of a CPP more than 3 months prior to the diagnosis. The underlying reason for unplanned admission is not transparent within the data, and the registered contact could be caused by other reasons than cancer, e.g. injury. Thus, the definition of RtD is defined contextually as opposed to based on the particular medical condition, yet, RtD is accepted as a relevant clinical marker for severity due to the poorer prognosis among patients diagnosed following an unplanned admission [3, 6]. The distribution of unplanned contacts caused by an emergency was similar in patients with and without psychiatric disorders (17 vs 18%, see result section). This could indicate that the higher probability of unplanned admission among patients with psychiatric disorders was not caused primarily by this group’s risk of e.g. injury or substance related emergencies [10, 14].

The large study population allowed us to stratify the analyses on both cancer types and subgroups of psychiatric disorders. However, overlaps were seen in the confidence intervals, particularly for unplanned admissions, which may have been related to low power for some of the cancer types.

The definition of pre-existing psychiatric disorders was based on previous work [24, 42, 43], as no standardised or internationally accepted method exists to assess general psychiatric disorders based on register data, and many psychiatric disorders are treated in primary care without hospital involvement. The inclusion of prescription medication made it possible to assess the impact on RtD from both psychiatric disorders that required involvement of a psychiatric department in secondary care and those primarily treated in general practice (and therefore no diagnosis was registered in the PCRR). However, it is not possible to determine the indication for prescription from the Danish National Prescription Registry, and it cannot be excluded that some of the included prescriptions were based on pre-diagnostic cancer symptoms such as inflammation rather than the psychiatric disorder. Sensitivity analysis, were prescription medication were excluded, did however not change the results markedly neither when different time frames were used. This indicates robust findings regardless of the definition used.

The stratified analysis focused primarily on CPP from primary care and unplanned admissions as research have found substantial difference in the cancer prognosis in these groups [3, 6]. However, the proportions of higher DCO in patients with psychiatric disorders were also interesting. Yet, due to a low sample in this group (*n* < 800), the analysis stratified on psychiatric subgroups would entail low statistical precision (e.g. with only 55 patients with anxiety and diagnosed through DCO).

### Results compared with existing literature

Previous studies have found that patients with dementia have higher probability of presenting as emergency [25]. To our knowledge, no studies have assessed the association between different pre-existing psychiatric disorders and RtD. For most of the assessed subtypes of psychiatric disorders, particularly severe disorders, the patients entered the diagnostic pathway more often through unplanned admissions and less often through CPPs from primary care even after adjustments psychiatric comorbidity. These results confirm the hypothesis of this study, but the explanations are likely to be complex. At the personal level, several factors in patients with psychiatric disorders may challenge the diagnostic process, including a lifestyle with high alcohol and substance abuse [44], little awareness of physical symptoms and difficulty comprehending messages from healthcare providers due to cognitive deficits [13, 15]. Feeling of hopelessness in depressed individuals has also been related to less motivation to act in general [15], which could delay healthcare seeking when experiencing symptoms. Further, research has found that patients with schizophrenia often experience healthcare discrimination, which may discourage these patients to seek care [13, 14]. Finally, acute challenges, such as delirium or psychosis, may challenge the diagnostic workup [14]. At the provider level, it has been proposed that symptoms from the psychiatric disorder can mask physical symptoms [14, 23], which in turn could delay or reduce the GP’s referral propensity. Finally, at the system level, research has found inferior quality of healthcare provided for patients with psychiatric disorders and challenges in coordinating care between healthcare sectors, which may lead to fragmented care for patients with severe mental disorders [12, 13]. Combinations of these factors may partially explain the higher risk of a less favourable RtD in patients with severe psychiatric disorders. One exception was patients with a stress disorder, who had a similar probability of being diagnosed through a CPP initiated in primary care or an unplanned admission as that of patients with no psychiatric disorders. This indicates that this subgroup of psychiatric disorders may differ from the other subgroups in terms of help-seeking and referral from primary care.

The results indicate that variations exist between cancer types in terms of the association between psychiatric disorders and RtD. We found that cancer types that are more likely to be diagnosed through a CPP initiated in primary care (e.g. breast cancer, melanoma), i.e. the so-called easy-to-diagnose cancer types [45, 46], showed fewer variations in RtD according to psychiatric disorders. Reversely, for more of the cancer types that are less likely to be diagnosed through a CPP initiated in primary care (e.g. pancreatic, lung, liver cancer), i.e. the so-called hard-to-diagnose cancer types [45, 46], we found a statistically significantly lower probability of being diagnosed through a CPP initiated in primary care in patients with psychiatric disorders compared to other patients. To some extent, these differences could be explained by the typical symptom presentation for these cancer types, which seems to be amplified in individuals with psychiatric disorders. The cancer types that are difficult to diagnose, e.g. due to unspecific symptom presentation, may also challenge the diagnostic work-up at individual, provider and system levels, which may result in lower recognition of bodily sensations, lower referral propensity and more acute presentations among patients with psychiatric disorders.

### Implications

In studies on cancer in general, low likelihood of being diagnosed after referral from primary care to an urgent cancer pathway and high likelihood of being diagnosed after an acute or emergency presentation have been linked with poor prognosis [3, 4]. Similar patterns were observed for patients with psychiatric disorders in this study, thus, based on this study, it could be hypothesised that focusing on the route to cancer diagnosis among patients with psychiatric disorders could be one strategy to improve the cancer survival in this group. Further research are also needed with particular focus of cancer diagnostics in primary care among patients with psychiatric disorders, as most cancer pathways is initiated in the primary sector [5] where challenges have been reported in the diagnostics of patients with psychiatric disorders [22, 23].

International literature suggests that challenges in the cancer pathway for this patient group are not limited to the RtD, which was illuminated in this study; their challenges also concern help-seeking behaviour, delays, advanced tumour stage at diagnosis, inferior treatment, survivorship and end-of-life care [13–16, 20, 21, 23]. Thus, as supported by other studies, there seems to be substantial potential to optimise the cancer pathway for patients with psychiatric disorders.

More research should focus on supportive measures, e.g. integrated care models, shared care, case managers [13] and multidisciplinary teams [14], to ensure better cancer pathways for these patient groups. Our findings suggest that there is room for improving the referral of patients with pre-existing psychiatric disorders to CPPs. More knowledge is needed on how the GPs can be supported in diagnosing cancer in this patient group as many appear to go undetected [13]. To target the efforts, more insight is also needed into whether special attention should be given to specific patients groups, e.g. patients with pre-existing psychiatric disorders who are socially deprived or have physical comorbidity.

## Conclusion

This register-based cohort study found that patients with pre-existing psychiatric disorders more often experience less favourable RtD. All three hypotheses were confirmed, i.e. that patients with pre-existing psychiatric disorders were more often diagnosed through less favourable routes than patients without, that patients with severe psychiatric disorders were more often diagnosed after unplanned admissions, and that the association between pre-existing psychiatric disorders and RtD varied with cancer type. Low CPP referral from primary care was more pronounced for many hard-to-diagnose cancer types and severe psychiatric disorders. However, there were exceptions, such as patients with stress disorders.

This study underpins the literature reporting that patients with pre-existing psychiatric disorders are more likely to experience substantial challenges in the cancer pathway. Research is needed to help both the patients and the healthcare providers ensure optimal diagnostics of cancer in this patient group.

## Supplementary Information

**Additional file 1: Part 1**. Distribution of cancer types. **Part 2**. Distribution of RtD according to different definitions of pre-existing psychiatric disorder (*n* = 155,851). **Part 3**. Relative risk ratio* for presenting in each route compared with “CPP – primary care” according to different definitions of pre-existing psychiatric disorders. **Part 4**. Estimated probability* of presenting through “CPP – primary care” and “unplanned admission” for each cancer type according to pre-existing psychiatric disorders based on hospital diagnosis registration.

## Data Availability

All data is assessed and provided through Statistics Denmark [29, 31–37]. Data is closed for public access and it is not possible for the authors to share the raw data because all data are stored only at Statistics Denmark according to the Danish regulations of research and can only be accessed by remote access VPN. Statistics Denmark does not allow to subtract data from Statistics Denmark which can identify any included patient. Therefore, no data can be shared from this project.

## Abbreviations

ATC: Anatomical Therapeutic Chemical
CCI: Charlson’s Comorbidity Index
CI: Confidence intervals
CPP: Cancer patient pathway
DCO: Death certificate only
DNPR: Danish National Patient Registry
GP: General practitioner
ICD-10: International Classification of Diseases, 10th version
ISCED: International Standard Classification of Education
MLR: Multinomial logistic regression
PCRR: Danish Psychiatric Central Research Register
RRR: Relative risk ratio
RtD: Route to diagnosis
SD: Standard deviation
TWW: Two-week wait
