# Supplementary material for: Routes to cancer diagnosis for patients with pre-existing psychiatric disorders: a nationwide register-based cohort study
Source: BMC Cancer. 2022 Apr 29;22:472. doi: 10.1186/s12885-022-09598-x (PMC9055745; doi:10.1186/s12885-022-09598-x)
Supplement: Supplementary file 1 — Additional file 1: Part 1. Distribution of cancer types. Part 2. Distribution of RtD according to different definitions of pre-existing psychiatric disorder (n = 155,851). Part 3. Relative risk ratio* for presenting in each route compared with “CPP – primary care” according to different definitions of pre-existing psychiatric disorders. Part 4. Estimated probability* of presenting through “CPP – primary care” and “unplanned admission” for each cancer type according to pre-existing psychiatric disorders based on hospital diagnosis registration. [file 12885_2022_9598_MOESM1_ESM.docx]

**Appendix**

Part 1. Distribution of cancer types

| **Cancer type** | **ICD-10** | **Total** | | |
| --- | --- | --- | --- | --- |
|  |  | **N** | | **%** |
| Head and neck | C00-C149 | 4,110 | (2.6) | |
| Oesophagus | C15 | 2,285 | (1.5) | |
| Stomach | C16 | 2,673 | (1.7) | |
| Colon | C18 | 15,102 | (9.7) | |
| Rectum | C29 | 7,231 | (4.6) | |
| Liver | C22 | 2,037 | (1.3) | |
| Pancreas | C25 | 4,327 | (2.8) | |
| Lung | C34 | 19,482 | (12.5) | |
| Malignant melanoma | C43 | 11,092 | (7.1) | |
| Breast | C50 | 22,181 | (14.2) | |
| Uterus | C54 | 3,535 | (2.3) | |
| Ovary | C56-C57 | 2,274 | (1.5) | |
| Female genitals | C51-C53, C55 | 2,368 | (1.5) | |
| Prostate | C61 | 20,846 | (13.4) | |
| Male genitals | C60, C62-C639 | 1,740 | (1.1) | |
| Kidney | C64-C59 | 4,276 | (2.7) | |
| Bladder | C67 | 3,827 | (2.5) | |
| Eye, brain, CNS | C69-C719 | 2,558 | (1.6) | |
| Endocrine glands | C73-C749 | 1,698 | (1.1) | |
| Lymphoma | C81-C859 | 5,842 | (3.8) | |
| Multiple myeloma | C90 | 2,145 | (1.4) | |
| Leukaemia | C89-C959 | 4,073 | (2.6) | |
| Other | All other C codes | 10,149 | (6.5) | |

*CNS: central nervous system*

Part 2. Distribution of RtD according to different definitions of pre-existing psychiatric disorder (n=155,851)

|  | **Routes to diagnosis** | | | | | | | | | | | | | | | | | |
| --- | --- | --- | --- | --- | --- | --- | --- | --- | --- | --- | --- | --- | --- | --- | --- | --- | --- | --- |
|  | DCO | | Screening | | CPP – primary care | | CPP – secondary care | | Unplanned admission | | Elective – other | | Outpatient – other | | Unknown | |  |  |
|  | % | | | 95% CI | % | 95% CI | % | 95% CI | % | 95% CI | % | 95% CI | % | 95% CI | % | 95% CI | % | 95% CI |
| **Pre-existing psychiatric disorder up to 2 years prior to cancer diagnosis*** | | | | | | | | | |  |  |  |  |  |  |  |  |  |
| No (n=132,082) | 0.3 | | | (0.3-0.3) | 7.9 | (7.8-8.1) | 45.7 | (45.2-45.8) | 18.8 | (18.6-19.0) | 14.3 | (14.1-14.5) | 1.1 | (1.1-1.2) | 7.0 | (6.9-7.2) | 5.0 | (4.9-5.1) |
| Yes (n=23,769) | 1.6 | | | (1.4-1.7) | 6.1 | (5.8-6.4) | 37.2 | (36.6-37.8) | 20.6 | (20.1-21.1) | 22.3 | (21.8-22.9) | 1.4 | (1.3-1.6) | 6.3 | (6.0-6.6) | 4.4 | (4.1-4.6) |
|  |  | | |  |  |  |  |  |  |  |  |  |  |  |  |  |  |  |
| **Pre-existing psychiatric disorder up to 10 years prior to cancer diagnosis**** | | | | | | | | | |  |  |  |  |  |  |  |  |  |
| No (n=124,662) | 0.3 | | | (0.3-0.3) | 8.0 | (7.8-8.1) | 45.7 | (45.5-46.0) | 18.7 | (18.4-18.9) | 14.1 | (13.9-14.3) | 1.1 | (1.1-1.2) | 7.0 | (6.9-7.1) | 5.0 | (4.9-5.1) |
| Yes (n=31,189) | 1.4 | | | (1.2-1.6) | 6.2 | (5.9-6.4) | 38.2 | (37.6-38.7) | 20.6 | (20.2-21.0) | 21.2 | (20.8-21.7) | 1.4 | (1.3-1.5) | 6.5 | (6.3-6.8) | 4.4 | (4.2-4.7) |
|  |  | | |  |  |  |  |  |  |  |  |  |  |  |  |  |  |  |
| **Pre-existing psychiatric disorder up to 5 years prior to cancer diagnosis, excluding “prescription-based mental disorders” ***** | | | | | | | | | | | | | | | | |  |  |
| No (n=142,161) | 0.4 | | | (0.3-0.4) | 7.8 | (7.7-8.0) | 45.1 | (44.8-45.4) | 20.0 | (18.8-19.2) | 14.6 | (14.4-14.8) | 1.1 | (1.0-1.2) | 7.0 | (6.8-7.1) | 5.0 | (4.9-5.1) |
| Yes (n=13,690) | 1.7 | | | (1.5-1.9) | 5.5 | (5.2-5.9) | 35.1 | (34.3-35.9) | 20.3 | (19.6-20.9) | 24.9 | (24.2-25.6) | 1.5 | (1.4-1.8) | 6.4 | (5.9-6.8) | 4.5 | (4.2-4.9) |
|  |  | | |  |  |  |  |  |  |  |  |  |  |  |  |  |  |  |

*patients in contact with the secondary healthcare system due to one of the included psychiatric disorders within ***two*** ***years*** of the cancer diagnosis or identified according to the category “prescription-based mental disorders”

**patients in contact with the secondary healthcare system due to one of the included psychiatric disorders within ***ten*** ***years*** of the cancer diagnosis or identified according to the category “prescription-based mental disorders”

******excluding*** patients in the category “prescription-based mental disorders” to assess the impact of psychiatric disorders on RtD based on diagnoses based on registered hospital contacts only

Part 3. Relative risk ratio* for presenting in each route compared with “CPP – primary care” according to different definitions of pre-existing psychiatric disorders

| **Definition of pre-existing psychiatric disorders** | | | | | | |
| --- | --- | --- | --- | --- | --- | --- |
|  | Pre-existing psychiatric disorder up to **2** years prior to cancer diagnosis | | Pre-existing psychiatric disorder up to **10** years prior to cancer diagnosis | | Pre-existing psychiatric disorder up to **5** years prior to cancer diagnosis, **excluding** “prescription-based mental disorders” | |
| **Routes to diagnosis** | RRR | (95% CI) | RRR | 95% CI | RRR | 95% CI |
| **DCO** |  |  |  | | |  |
| Any psychiatric disorder |  |  |  |  |  |  |
| No | 1 |  | 1 |  | 1 |  |
| Yes | **3.61** | **(3.02-4.31)** | **3.49** | **(3.00-4.06)** | **3.12** | **(2.47-3.94)** |
| **Screening** |  |  |  |  |  |  |
| Any psychiatric disorder |  |  |  |  |  |  |
| No | 1 |  | 1 |  | 1 |  |
| Yes | 0.91 | (0.82-1.01) | 0.90 | (0.78-1.03) | 0.97 | (0.81-1.15) |
| **CPP** – **primary care** | | | | | | |
| Any psychiatric disorder |  |  |  |  |  |  |
| No | - | - | **-** | **-** | **-** | **-** |
| Yes | **-** | **-** | **-** | **-** | **-** | **-** |
| **CPP** – **secondary care** |  |  |  |  |  |  |
| Any psychiatric disorder |  |  |  |  |  |  |
| No | 1 |  | **1** |  | **1** |  |
| Yes | **1.17** | **(1.06-1.29)** | **1.13** | **(1.02-1.26)** | **1.16** | **(1.02-1.34)** |
| **Unplanned admission** | | | | | | |
| Any psychiatric disorder |  |  |  |  |  |  |
| No | 1 |  | **1** |  | **1** |  |
| Yes | **1.47** | **(1.35-1.60)** | **1.36** | **(1.23-1.50)** | **1.62** | **(1.42-1.84)** |
| **Elective – other** | | | | | |  |
| Any psychiatric disorder |  |  |  |  |  |  |
| No | 1 |  | 1 |  | 1 |  |
| Yes | **1.33** | **(1.13-1.58)** | 1.23 | (1.02-1.47) | 1.31 | (1.04-1.64) |
| **Outpatient – other** | | | | | |  |
| Any psychiatric disorder |  |  |  |  |  |  |
| No | 1 |  | 1 |  | 1 |  |
| Yes | 1.07 | (0.99-1.15) | 1.07 | (0.98-1.18) | 1.09 | (0.96-1.24) |
| **Unknown – other** | | | | | |  |
| Any psychiatric disorder |  |  |  |  |  |  |
| No | 1 |  | 1 |  | 1 |  |
| Yes | 1.15 | (0.96-1.38) | 1.14 | (0.97-1.34) | 1.19 | (0.88-1.61) |

**Adjusted for sex, age, year of diagnosis, comorbidity, education, ethnicity, cohabitation, region of residence and cluster robust standard errors to account for effect of clustering of observations around cancer diagnoses*

*CPP: cancer patient pathway, DCO: death certificate only*

Part 4. Estimated probability* of presenting through “CPP – primary care” and “unplanned admission” for each cancer type according to pre-existing psychiatric disorders ***based on hospital diagnosis registration.***

*Adjusted for sex, age, year of diagnosis, comorbidity, education, ethnicity, cohabitation and region of residence
*CPP: cancer patient pathway, CNS: central nervous system*
